# Ebola response in Sierra Leone: The impact on children

**DOI:** 10.1016/j.jinf.2016.04.016

**Published:** 2016-07-05

**Authors:** Felicity Fitzgerald, Waheed Awonuga, Tejshri Shah, Daniel Youkee

**Affiliations:** a Infection, Immunity, Inflammation and Physiological Medicine, UCL Institute of Child Health, UK; b Kerry Town Ebola Treatment Centre, Save the Children International, Sierra Leone; c Live Case Management Team, Western Area Emergency Response Centre, Freetown, Sierra Leone; d Department of Paediatric Infectious Diseases, Imperial College NHS Healthcare Trust, UK; e Kings Sierra Leone Partnership, Connaught Hospital, Freetown, Sierra Leone

**Keywords:** Ebola virus disease, Children, Paediatrics, Viral haemorrhagic fever

## Abstract

The West African Ebola virus disease (EVD) outbreak is the largest ever seen, with over 28,000 cases and 11,300 deaths since early 2014. The magnitude of the outbreak has tested fragile governmental health systems and non-governmental organizations (NGOs) to their limit. Here we discuss the outbreak in the Western Area of Sierra Leone, the shape of the local response and the impact the response had on caring for children suspected of having contracted EVD. Challenges encountered in providing clinical care to children whilst working in the “Red Zone” where risk of EVD is considered to be highest, wearing full personal protective equipment are detailed. Suggestions and recommendations both for further research and for operational improvement in the future are made, with particular reference as to how a response could be more child-focused.

## Introduction

The West African Ebola virus disease (EVD) outbreak is the largest ever seen, with over 28,000 cases and 11,300 deaths since early 2014.^1^ The magnitude of the outbreak has tested fragile governmental health systems and non-governmental organizations (NGOs) to their limit. Spread from a rural outbreak source in the Guéckédou district of Guinea to the crowded urban environments of Monrovia, Conakry and Freetown allowed viral transmission on an unprecedented scale.^2^ Over a year after the EVD transmission was established in Freetown, Sierra Leone, it is time to take stock. At the peak of the outbreak, pragmatic decisions were taken about how to respond to the novel situation of EVD transmission in impoverished urban settings. Without understanding those decisions and the shape of the response in Sierra Leone, it is difficult to interpret data emerging from the outbreak.

Here we describe the setting of the outbreak and the emergency response in the densely populated Western Area of Sierra Leone including the capital Freetown, to demonstrate features of the response which particularly impacted on children. Issues affecting data collection will be described, followed by an urgent call for granular and contextual research into the current outbreak in order to plan better for the future.

### Ebola in the Western Area of Sierra Leone

The Western Area of Sierra Leone is the most densely populated area of Sierra Leone, with ∼1 million inhabitants at the last published census (2004), over 25% of whom live in informal housing, and only 26% of whom have a private toilet.^3^ In certain areas of Freetown, the population density reaches 40,000 persons/km^2^.^3^ In 2013, Sierra Leone had 0.2 doctors and 1.7 nurses/midwives per 10,000 population.^4^ For context, corresponding figures in South Africa were 7.8 and 49, and 27.9 and 88.3 in the United Kingdom.^4^ In this environment of overcrowding, poor sanitation and an overstretched health system, transmission of EVD was the most intense out of all areas affected in this outbreak.^5^

Ebola virus is an enveloped filovirus thought to have a natural host in bats.^6^ It has previously caused isolated outbreaks in Uganda and the Democratic Republic of Congo with a mortality rate of up to 81%.^7^, ^8^ Transmission is via contact with bodily fluids particularly in later stages of the disease, with corpses being highly contagious. The incubation period is 2–21 days (mean 11.4).^9^ Pooled adult and paediatric data as well as clinical experience suggest that clinical manifestations of the disease in this outbreak differ from previous ones. Along with fever and fatigue, gastrointestinal features are much more prominent, occurring in 50–68% of cases with diarrhoea being significantly associated with a fatal outcome.^10^, ^11^, ^12^ Gastrointestinal fluid loss and resultant electrolyte imbalance can be dramatic with up to 10 L of diarrhoea a day and resultant hyponatraemia and hypokalaemia.^13^, ^14^ Spontaneous bleeding is uncommon in this outbreak, seen in 2–18% of patients.^9^, ^10^, ^15^ Overall, the mortality rate for this outbreak is ∼56%.^16^

### The Ebola holding unit model

One of the most insidious and destructive features of EVD is its proclivity for infecting health care workers (HCWs). To date, over 880 HCWs have been infected in this outbreak with a mortality rate of 58%.^1^ Coupled with the non-specificity of symptoms, this breeds distrust within hospitals and other health care facilities whereby doctors and nurses view their patients with fear and in turn patients fear health facilities. Without a robust system for triaging and testing potential EVD patients, hospitals closed as staff were too afraid to come to work. Such a situation was seen in the John F Kennedy hospital in Monrovia, Liberia in July 2014.^17^ The impact this closure had on nationwide mortality rates from other conditions is not fully defined. In Sierra Leone, inpatient admissions dropped by 70% from May to October, leaving a predicted 35,000 patients without essential inpatient care,^18^ and additional deaths from malaria alone exceeding those from EVD.^19^, ^20^ A reduction in vaccine coverage led to a prediction of 12,000 additional deaths from measles.^21^ In order to avoid a catastrophic cessation of any health care provision in the Western Area, a novel Ebola Holding Unit (EHU) model was developed.^22^

**Figure 1:**
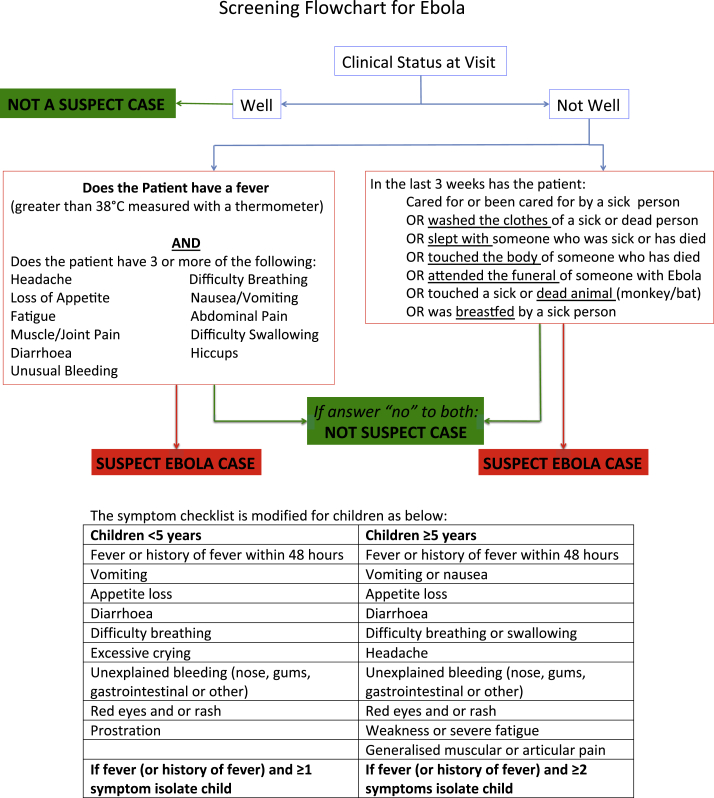
Screening Flowchart for Ebola used on attendance at health care facilities in the Western Area of Sierra Leone.

In the EHU model, the most frequented health care facilities – hospitals and community health centres – all have an associated EHU on site for safe isolation and testing of potential EVD patients. All those seeking health care are screened at the gate using a questionnaire asking about potential EVD contacts and symptoms (Fig. 1). Potential Ebola suspect cases are also referred by district surveillance officers (DSOs) in the community, or from observational interim care centres (OICCs). OICCs care for, and observe, asymptomatic children who have had close contact with an EVD patient and have no other caregiver for the 21-day incubation period of EVD.

**Figure 2:**
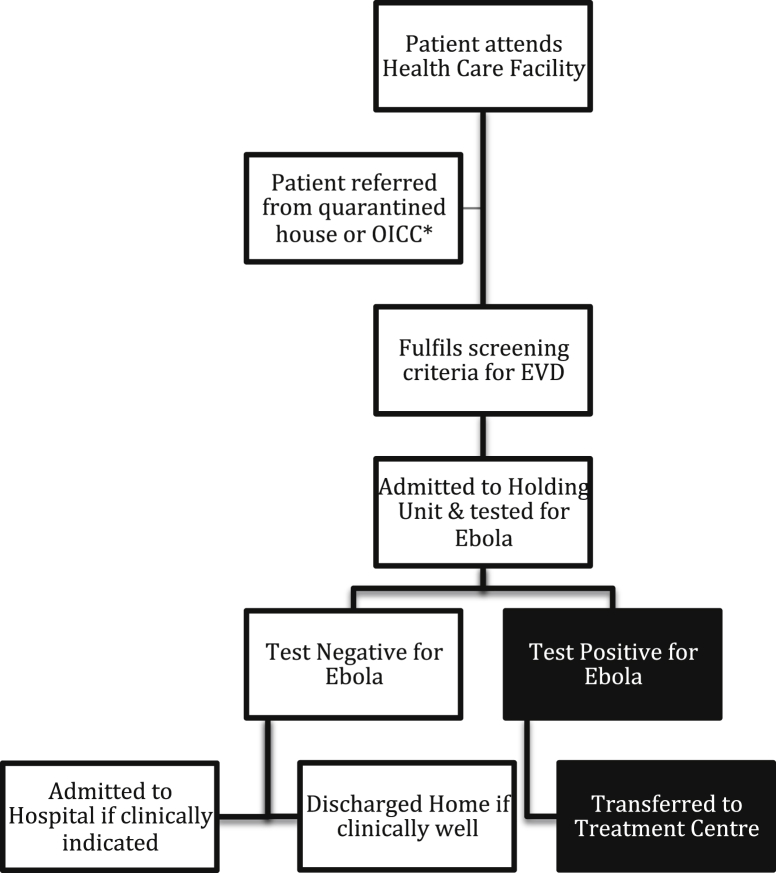
Flowchart of patients attending health care facilities in Freetown, Sierra Leone. *OICC: Observational Interim Care Centre.

Those with a positive contact, or a fever and 3 or more symptoms consistent with EVD (Fig. 1), are admitted as a suspected case to the EHU for EVD testing (Fig. 2). A venous blood sample is sent to specialist laboratories with category 4 facilities where a quantitative polymerase chain reaction (PCR) for Ebola is performed. Depending on the test result, patients are transferred on to an Ebola treatment centre (ETC: these tended to be large specially built and designed units set up and run by NGOs in response to the outbreak), admitted to the general ward if still needing inpatient treatment for a non-EVD condition, or discharged home if recovered. In Freetown, patient movement was coordinated by the Western Area emergency response centre (WAERC), which was in daily contact with each EHU and ETC, community DSOs and the laboratories processing samples for EVD testing.

The principal features of EHUs were that they were rapidly built (with a lead time of as little as 48 h); co-located at existing hospitals and functioned in partnership between an NGO and the Sierra Leonean Ministry of Health.^22^ Their primary functions were threefold: isolation of suspected cases to minimize community transmission, maintenance of non-EVD-related healthcare provision, and the creation of an EVD-free environment within the adjoining hospital through gate and ward daily screening combined with immediate on site isolation for protection of healthcare workers and other patients.^23^ In our experience, the provision of care across EHUs was heterogeneous, dependent on staffing numbers, skillsets and the space available within the facility. Care varied between a “no needle” policy with provision of oral medications only, to more sophisticated therapy with intraosseous infusions and blood transfusions.

**Figure 3:**
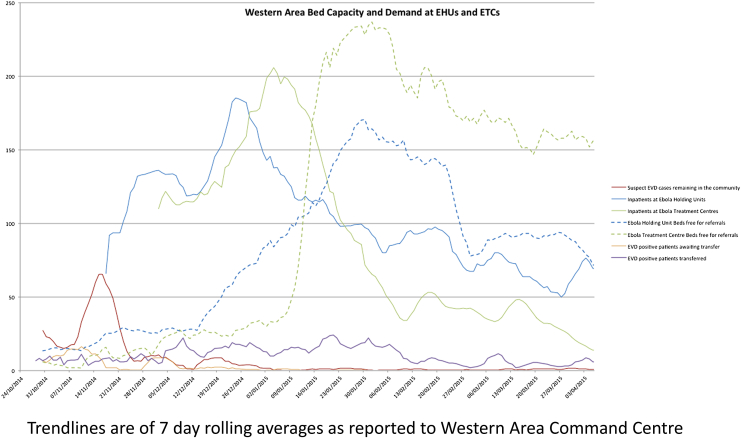
Graph of Western Area Bed Capacity and Demand at Ebola Holding Units and Ebola Treatment Centres. Trendlines are of 7 day rolling averages as reported to the Western Area Ebola Response Command Centre.

At the peak of the Sierra Leone outbreak in late 2014, there were bottlenecks at each stage of this system. Patients could wait for 48 h to be admitted to an EHU, up to 8 days in an EHU (the mean duration of admission in late November 2014 was 2.3 days) waiting for test results or bed availability in an ETC, and then face long transfer distances to the nearest available ETC bed. During the peak of the outbreak laboratories were not co-located at EHUs and laboratory capacity did not meet demand, so the mean turnaround time for results was 48 h. For example, in the week ending 18th November 2014, EHU bed capacity stood at 130, and the Western Area ETC bed capacity at 126, all of which were occupied (Fig. 3). 486 suspected cases reported in the community were unattended for >24 h as the EHUs were full. There were 158 new EVD cases diagnosed of which 44 (28%) had to be transferred at least 400 km to the nearest free ETC bed (WAERC data, unpublished). By early February 2015, there were 619 Western Area ETC beds of which 32 were occupied (5%). Ambulance availability varied between 4 and 30 per day for the whole Western Area, which also hindered efficient patient flow.

These bottle necks did not only delay access to ETCs which were often better staffed and better equipped to manage EVD patients, but also increased the time during which those who subsequently tested negative were exposed to those who did have EVD. The risk of EHUs acting as an amplification site for EVD has been raised, although positive readmission rates at EHUs in Freetown of 1.5–3.3% compare favourably to previously documented rates of 9% in other regions.^24^, ^25^ Notably all the readmitted patients documented by Fitzpatrick et al.^24^ had exposure to EVD in the community before their first admission.

### Holding units and children

The WHO case definition for suspected EVD in children is even broader than for adults.^26^ Children under 5 years of age only need to have one qualifying symptom to be admitted to an EHU, meaning that large numbers of children will be admitted to subsequently test negative.^26^ This proportion increased as EVD cases fell: in late November 2014 13/39 (33%) of children admitted at the main children's hospital EHU tested positive, whereas in late April 2015 73 children were admitted with none testing positive (WAERC data, unpublished). There are currently no data available on the impact of admission to an EHU for children who subsequently test negative for EVD. Both the potential risk of nosocomial EVD transmission and the impact on mortality for common conditions such as malaria or sepsis are currently unquantified.

Due to the perceived risk of EVD exposure, most EHUs adopted a policy of forbidding asymptomatic mothers from accompanying their unwell children into EHUs. This, coupled with the fact that many children had already lost family members to EVD, meant that approximately 40% of children were admitted unaccompanied to EHUs (Fitzgerald et al. under review). Our team found the provision of care to unaccompanied children in a Red Zone (full barrier nursing, where risk of Ebola exposure was judged to be highest) environment extremely challenging.

The first challenge was the difficulty of personal protective equipment (PPE). The occlusive heat of PPE means time in the Red Zone is limited, and must be divided between duties such as cleaning, drug administration, admitting and discharging patients as well as support for activities of daily living. At the peak of the outbreak estimates of time available for patient care and medical decision-making varied between 3 and 20 min per patient.^27^, ^28^ Attempting to give sufficient oral rehydration solution (ORS) to a sick child alongside other medications in this time frame is almost impossible. Furthermore, in combination with a language barrier experienced by most international workers, PPE hinders effective communication with a child as it makes any worker look terrifying. Finally, PPE is flimsy: it will not withstand children's prying fingers, risking breaches and making close interaction dangerous.

Secondly, ambulant unaccompanied children are difficult to control. The key to a safe EHU is isolation and patient separation, a lonely child is liable to leave their bed space to seek comfort from other patients in the unit, increasing the risk of cross-contamination in the unit. Non-ambulant children in cots would cry so piteously that other patients with as yet unconfirmed EVD status would take them out and comfort them, again increasing the risk of EVD transmission.

Thirdly, Red Zones are full of risks beyond EVD exposure, including sharps bins and buckets of strong chlorine solution that children may mistake for a bath or attempt to drink, both situations that have been experienced in both our units and others in Freetown (pers. comm., T Helderman, Medair). We have also had experience of children trying to escape out of the Red Zone and back to their parents.

One unit did insist on children being accompanied by caregivers despite the risk of EVD due to staffing constraints. The differential impact on outcome of being admitted with a caregiver or not has not been investigated, nor has the risk to caregivers been quantified.

Finally, there is the question of transfer distances. Even the closest ETCs were 45–60 min drive from most of Freetown's EHUs, which many children would have to undergo alone as minimal clinical care was provided in the ambulances. For the longer transfer distances, mortality rates for adults and children of up to 60% in the ambulance were reported back to our units.

### Data availability

There are many pressing questions about whether potentially modifiable factors such as caregiver accompaniment and transfer distances, not to mention medical care given, affected mortality in children admitted to EHUs. However effective data collection is not straightforward. Linking data from EHUs and ETCs is critical. Patients may have spent several days in an EHU receiving care prior to transfer. Without EHU data, results from an ETC alone will be subject to a survivor bias that may result in dramatically lower mortality rates than that seen elsewhere.^10^, ^28^, ^29^, ^30^, ^31^ Similarly, data from EHUs alone will be missing outcomes for patients transferred out.^15^ Data from ETCs was not routinely fed back to the WAERC, the national Ebola response centre or to the centralized viral haemorrhagic fever (VHF) database curated by the CDC, meaning that larger scale epidemiological studies are missing outcomes in up to 50% of confirmed EVD cases.^11^, ^32^ This meant the live case management team at the WAERC were unable to track patients through to their final outcomes in ETCs, and so could not even inform relatives of a patient's death or survival, particularly if the patient had been transferred out of area.

### Future research and recommendations

There is urgent need for a detailed investigation of the trajectory of both adults and children admitted to EHUs. It is crucial to investigate the impact of the EHU model on those confirmed to have EVD and those who subsequently test negative. The impacts on mortality from diseases other than EVD in EHUs have so far only been modelled rather than directly investigated. There are potentially modifiable health system factors such as caregiver accompaniment or transfer distances that could impact on mortality. Alongside this, the risk for caregivers of nosocomial infection must be quantified. Even if the risk of admission to an EHU is deemed unacceptably high, an evidence base could be used to lobby for employing EVD survivors (understood to be at low risk of reinfection) to look after children admitted to EHUs, as has happened in limited circumstances.^33^

The EHU model was set up as a pragmatic, rapid response to a health care crisis when existing facilities were overwhelmed. EHUs effectively protected hospitals and their staff from EVD infection and isolated several thousand EVD patients with minimal staff and resources.^15^, ^22^ Confines of space and resources necessitated transferring out confirmed EVD patients to ETCs which may have impacted on mortality due to the transfer itself. In the future, a model whereby at the peak of an outbreak, suspect patients are immediately transferred to dedicated ETCs for both testing and treatment might mitigate both the duration of time spent in a unit for those who test negative, and delays in instituting the more invasive and aggressive care that tended to be available in better-resourced ETCs. However this requires excellent channels of communication and ready availability of ambulances, alongside ETC capacity, which were in scant supply in Freetown until December 2014 (Fig. 3).

It is crucial that any model of care should be responsive and as necessary adapted over the course of the outbreak. Although rapid transfer with testing remote from general healthcare facilities is appropriate at the peak of an outbreak when prevalence of EVD in an EHU is high, this is unfeasible at the end, when most are suffering from non-EVD complaints. As discussed above, this is particularly true for children given the breadth of the case definition. Refinement and adaption of the case definition according to emerging evidence and local disease prevalence should be encouraged. For example, the stratification of the case definition into high or low risk for EVD is an important operational distinction that could facilitate rapid patient referral to the most appropriate centre of care.

A transformative intervention would be a sensitive and specific point of care (POC) EVD test that could avoid the delays inherent in transporting blood samples to offsite laboratories for testing. A POC test would negate the need for lengthy admissions in EHUs and so minimize unnecessary exposures to EVD for those testing negative. Several of these tests are in development with promising results in adults, although few data are available from children.^34^, ^35^

Availability of effective vaccination would completely alter the management of EVD outbreaks in the future, and recent evidence looks promising.^36^, ^37^ Both a recombinant chimpanzee adenovirus vector (recombinant chimpanzee adenovirus type 3-vectored Ebola Zaire vaccine (ChAd3-EBO-Z)) and a recombinant vesicular stomatitis virus vector (recombinant, replication-competent vesicular stomatitis virus-based candidate vaccine expressing Zaire Ebolavirus glycoprotein (rVSV-ZEBOV)) have reached Phase III trials^36^ (ClinicalTrials.gov Identifier NCT02485301). In a cluster-randomised ring vaccination trial in Guinea, rVSV-ZEBOV was found to have 100% efficacy (95% CI 74.7–100) with a 75.1% effectiveness (95% CI 7.1–94.2) for all eligible adults at the cluster level.^36^ However, it should be noted that there are minimal safety or immunogenicity data in children: a crucial knowledge gap that must be rapidly addressed. Breakthrough EVD cases occurred in under eighteen year olds (excluded from the trial) in both the immediate and delayed groups of the ring vaccination trial highlighting that effective adult vaccination may not be sufficient to protect unvaccinated children.^36^ Two trials in children are currently planned but not yet recruiting (ClinicalTrials.gov Identifiers NCT02548078 and NCT02509494).

## Conclusions

The EHU model was a major contributor to the effectiveness of the Ebola Response in Sierra Leone, isolating the majority of suspect cases in the Western Area of Sierra Leone.^22^ Questions remain regarding the impact of the model on patients with and without EVD, and how the response might be improved in future. These questions will be unanswered without a rapid and focused effort to collect data from EHUs, ETCs, staff members and relatives, and to triangulate these with laboratory results, burial databases and child protection data. In the September 2015 reports from the Sierra Leonean national Ebola response centre, there are still 1500 patients with confirmed EVD with an unknown outcome. Our understanding of EVD outcomes is poor, and risks remaining so without urgent collation and sharing of the data available. It is only with a more comprehensive evidence base that we will be able to move forward with successful implementation of interventions such as vaccines or POC tests. At present, there is still no consensus on critical questions such as the safety of caregiver accompaniment of children in a Red Zone. Data sharing between NGOs and government organisations will be key to creating a safer and more effective Ebola response in the future, both for children, adults and the health care workers that staff them.

## Conflicts of interest

We declare no conflicts of interest. Felicity Fitzgerald is funded by a grant from the Medical Research Council (MR/K023535/1). No other funding was received for this work.

## Figures and Tables

From *Clinical Management of patients in the Ebola treatment Centres and other Care Centres in Sierra Leone: A Pocket Guide*. Interim emergency guidelines. Sierra Leone adaptation. World Health Organization. December 2014.
